# Synthesis and characterization of Ni-MOF and CoFe_2_O_4_/Ni-MOF as reusable heterogeneous catalysts for the synthesis of 5-substituted 1*H*-tetrazole

**DOI:** 10.1038/s41598-025-23799-x

**Published:** 2025-11-17

**Authors:** Madeh Faizy, Somayeh Molaei, Mohammad Ghadermazi

**Affiliations:** Department of Chemistry, Faculty of Science, University of Kurdistan, Sanandaj, Iran

**Keywords:** Nickel, 2,6-Pyridinedicarboxylic acid, Ni-MOF, CoFe_2_O_4_/Ni-MOF, Synthesis of tetrazole, Heterogeneous catalysis, Green chemistry, Inorganic chemistry

## Abstract

The purpose of this study is to describe the synthesis of typical porous metal-organic framework (MOF) materials based on nickel 2,6-pyridinedicarboxylic acid (Ni-MOF). Also, using a simple in-situ solvothermal method, CoFe_2_O_4_/nickel 2,6-Pyridinedicarboxylic acid (CoFe_2_O_4_/Ni-MOF) was synthesized as a magnetic MOF composite. The CoFe_2_O_4_/Ni-MOF and Ni-MOF were characterized by powder X-ray diffraction (XRD), field emission scanning electron microscopy (FE-SEM), energy-dispersive X-ray spectrometer (EDS), vibrating-sample magnetometer (VSM), Fourier transform infrared spectroscopy (FTIR), and nitrogen adsorption − desorption isotherm to confirm successful integration and its porous, and magnetic nature. The potential of Ni-MOF and CoFe_2_O_4_/Ni-MOF as catalysts was investigated for the one-pot synthesis of a diverse array of pharmaceutically active 5-substituted 1*H*-tetrazole derivatives. To optimize the reaction conditions, the efficiency of catalyst amounts, various solvents, and different temperatures has been studied using benzonitrile (1 mmol), a catalyst, and NaN_3_ (1.2 mmol) as a model reaction. The developed material demonstrated remarkable efficiency (80%) in the synthesis of 5-(phenyl)-1*H*-tetrazole. An important aspect of this strategy is that it adheres to ecologically favorable reaction conditions (The reactions are based on green chemistry under mild reaction conditions in H_2_O/EtOH solvent, at a relatively low temperature compared to the reported work). The CoFe_2_O_4_/Ni-MOF composite significantly outperformed the pure Ni-MOF, achieving an 80% yield in 20 min versus 40 min for the Ni-MOF, using the same catalyst mass (80 mg) in H_2_O /EtOH (1:1) solvent. The research demonstrates the deliberate design of a high-performance, retrievable magnetic MOF catalyst for sustainable organic synthesis.

## Introduction

Because their atomic orbitals are not fully labeled, transition metals (TMs) have a strong attraction for forming a wide variety of complexes with different organic ligands. Several reports have synthesized transition metal complexes in chelation with unique organic ligands. These ligands have a wide range of other uses as homogenous catalysts in various organic transformations^1–3^. Catalyst isolation and reusability are important issues for environmental sustainability. Because of their ease of handling, thermal stability, ease of isolation, and reusability, the heterogenization of the TM complexes through immobilization over various solid supports, such as magnetic nanoparticles^4^, silica^5^, zeolites^6^, carbon nanotubes^7^, and layered materials^8^, has thus been thoroughly investigated. Metal oxides are among the most widely used compounds because of their distinctive properties that can be developed. One of the most important methods used to improve these properties is mixing with other oxides, heteropolyacids, sulfate, phosphate, molybdate, tungstate, and others^9–11^.

Metal-organic frameworks (MOFs) and metal-organic polyhedras (MOPs) have collectively enticed a matchless attraction in research fields all around the world. A novel family of porous coordination polymers known as metal-organic frameworks (MOFs) is created by the chemical interaction of inorganic (metal nodes) and organic (linkers) compounds^12,13^. Metal-organic frameworks are organic-inorganic hybrid crystalline compounds consisting of inorganic metallic clusters^14–16^, also referred to as nodes, that are connected by organic linker molecules, i.e., spacers^17,18^. In the past two decades, the utilization of MOFs as a heterogeneous catalyst has offered a connotation surge as MOFs have been deliberated upon as an environmentally friendly alternative for heterogeneous catalysis^19^. MOFs have gained popularity for their exceptional features, including large surface area, variable pore sizes, ordered crystalline structures, and permanent porosity^20–22^. MOFs have multiple applications, including catalysis^20,23,24^, drug delivery^25,26^, separation^27^, gas storage^28^, batteries^29^, and water splitting^30^. Besides, the metal-organic framework caught the researcher’s attention due to its flexible design, unique structure is beneficial for both homogeneous and heterogeneous electrocatalysts^31–35^.

In previous research, various MOFs have been studied for different applications. For example, A facile and easy technique for the synthesis of phosphotungstic acid (PTA) with different weight contents loaded on UiO-66 (Zr) as efficient and reusable catalysts for the preparation of coumarin and dihydropyrimidinones derivatives was reported^36^. Magnesium-based metal-organic framework (Mg-BTC) was successfully synthesized from the interaction between trismic acid (BTC) as a linker and magnesium metal clusters as a node. The improvement of the catalytic activity of the prepared MOF in the presence of graphene oxide and gold nanoparticles was achieved. The catalytic activities of the prepared composites were tested toward CO oxidation, and the stability of these composites was investigated^37^. Copper and chromium bi-metallic organic frameworks (MOFs) were synthesized by reaction of 1,4-benzene dicarboxylic acid as a linker with chromium nitrate and copper nitrate at different mole ratios as metal ion sources. The adsorption isotherms and kinetics study of methyl orange removal from water were investigated spectrophotometrically using the batch adsorption technique. Mixed-component metal-organic frameworks (Cux-Cr100-x–MOF) are a promising adsorbent that can be used to clean up textile wastes^38^. A series of sulfate-loaded Cu-BDC (SO42-/Cu-BDC) catalysts with different sulfate content (15–85 wt% %) of SO_4_^2−^ were prepared by the wet impregnation technique in aqueous solution for one day. The acidity of catalysts increases with the increase in sulfate content up to 50 wt%. %SO_4_^2−^/Cu-BDC. The correlation between catalytic activity and surface acidity holds for the synthesis of 7-hydroxy-4-methyl coumarin. Catalyst activity indicates that the activity for the synthesis of 7-hydroxy-4-methyl coumarin is related to the availability of Brønsted acid sites which in turn depends of the SO_4_^2−^ content, which means we can use these catalysts in green chemistry^39^. One-pot synthesis techniques for creating Cu/Cr-MOF nanocomposites with varying molar ratios of Cu and Cr, followed by pyrolyzing at 450 ◦C to prepare different MOF-D, were provided. A high-performance supercapacitor electrode was also successfully fabricated. Additionally, electrochemical measurements of all prepared composites were achieved by covering graphite sheet electrodes with a definite amount of the active materials, and their responses were measured in 2.0 M KOH as an electrolyte using electrochemical impedance spectroscopy (EIS) measurements, galvanostatic charging and discharging (GCD), and cyclic voltammetry (CV)^40^. Mixed component metal-organic frameworks (Cu_x_-Cr_100−x_–MOF) as an adsorbent material for removal of MO from aqueous solution. Sulfate loaded on Cu-BDC (SO_4_^2−^/Cu-BDC) was prepared, then modified by SO_4_^2−^ introduced in Cu-BDC. The catalytic activity of the resultant sulfated Cu-BDC composite was studied by carrying out resorcinol and ethyl acetoacetate in a free Bachmann condensation reaction, which gives a high yield from 7-hydroxy-4-methyl coumarin^41^.

MOF materials are appealing for their capacity to adjust pore size and shape, from microporous to mesoporous, by adjusting the inorganic moiety connectivity and organic linkers^42^. Owing to the many possible combinations of organic linkers and metal ions, a vast number of MOF structures, now up to more than 20000, have been reported so far. The control of MOF crystal size at the nanometer level results in MOF NPs whose properties are no longer determined by their inner surface only, but also by their outer surface properties, due to their high external surface-area-to-volume ratio^43–45^.

This article is intended to survey recent progress in the synthesis and reactions of tetrazoles, whose discovery dates back over a century. Tetrazoles are characterized by a five-membered, doubly unsaturated ring consisting of one carbon and four nitrogen atoms^46,47^. The Swedish chemist Bladin introduced and named a five-membered N-rich heterocycle with four N-donor centers “tetrazole”^48^. *N*-Donor tetrazole derivatives possess physicochemical features, such as good thermostability/chemical stability, abundant coordination modes, high dipole moment, high formation enthalpy, basicity, and acidity, as well as fluorescence/luminescent and magnetic behaviors, which make them widely utilized in both academic and industrial fields, namely in medicinal/biological chemistry, coordination chemistry, and materials science^49–51^.

Magnetic MOF composites, created by combining MOFs and magnetic material, are particularly appealing due to their ability to attract magnets and be collected and positioned using an external field. Magnetic MOFs not only allow for easy separation but also eliminate the requirement for labor-intensive high-speed centrifugation and filtration following post-application^52^. Another significant advantage is that it improves the chemical and thermal stability of the resultant composites.

Many homogeneous and heterogeneous catalysts were used to prepare tetrazoles. The results of some conversions of nitrile to tetrazole are summarized in Table 1. Despite abundant reports for the synthesis of tetrazole, most of these protocols exhibit some serious drawbacks, such as the use of harsh reaction conditions, high temperature, prolonged reaction duration, low product yield, use of organic solvents, hazardous metals, the inclusion of strong Lewis acids, expensive reagents, low recyclability, and low recoverability of the catalyst. Therefore, designing a highly efficient, cost-effective, eco-friendly protocol for accessing a series of 5-substituted 1H-tetrazoles is a very benign task. The CoFe_2_O_4_/Ni-MOF and Ni-MOF exhibit better reaction time and recyclability under mild conditions in a green solvent. At a relatively low temperature compared to the reported work, tetrazole derivatives are prepared in H_2_O /EtOH solvent with good efficiency. 

Herein, we report a novel strategy to address this by designing a magnetically functionalized metal-organic framework. For the first time, we describe the synthesis of a CoFe₂O₄/Ni-MOF nanocomposite and its application as a powerful heterogeneous catalyst for the synthesis of 5-substituted 1 H-tetrazoles. The novelty of this work lies in the creation of a synergistic catalytic system that combines the exceptional Lewis acidity of the Ni-MOF with the magnetic properties of CoFe₂O₄, enabling unparalleled catalytic performance coupled with effortless magnetic recovery and outstanding reusability. This approach effectively bridges the gap between high catalytic performance and practical catalyst handling, offering a promising and sustainable alternative to conventional catalytic methodologies.

The specific research gap addressed by this study: (1) Gap in utilizing Ni-MOF for tetrazole synthesis: While some MOFs (like Zn-, Cd-, or Cu-based) had been reported for this reaction, the application of a Nickel-based MOF (Ni-MOF) as a Lewis acid catalyst for the synthesis of 5-substituted 1 H-tetrazoles was potentially unexplored or underreported. The study addresses the gap in knowledge regarding the catalytic efficacy, stability, and reusability of a cost-effective Ni-MOF for this specific transformation. (2) The critical gap in catalyst separation and recovery: Even a good MOF catalyst still requires filtration or centrifugation for recovery. The gap was the lack of a designed catalyst that combines the high catalytic activity of a Ni-MOF with the magnetic properties of a spinel ferrite (CoFe₂O₄) for utterly simple separation using an external magnet. (3) The performance comparison gap: The study doesn’t just create a new material; it systematically addresses the question: “Does embedding magnetic nanoparticles within a MOF enhance, diminish, or have no effect on its catalytic performance compared to the pristine MOF?” The gap was a lack of direct, comparative studies between a pristine MOF and its magnetic composite for this reaction, evaluating both activity and reusability.

## Experimental

### Synthesis of CoFe_2_O_4_

To produce CoFe_2_O_4_ nanoparticles using the coprecipitation method, 50 mL of distilled water was used to dissolve 0.5 mmol of CoCl_2_∙2H_2_O powder and 1 mmol of FeCl_3_∙6H_2_O powder while gassed with nitrogen. The first solution was then gradually supplemented with 25 mL dissolved NaOH (1 g). The final solution was refluxed for three hours. After cooling the reaction to room temperature, the black precipitate was separated using a magnet. The resulting CoFe_2_O_4_ nanoparticles were subsequently washed with ethanol and dried at 60 °C^53^.

### Synthesis of Ni-MOF framework

In the solvothermal synthesis of MOF, some references that describe similar approaches to bimetallic MOF synthesized include^27,36,54–58^.

**Fig. 1:**
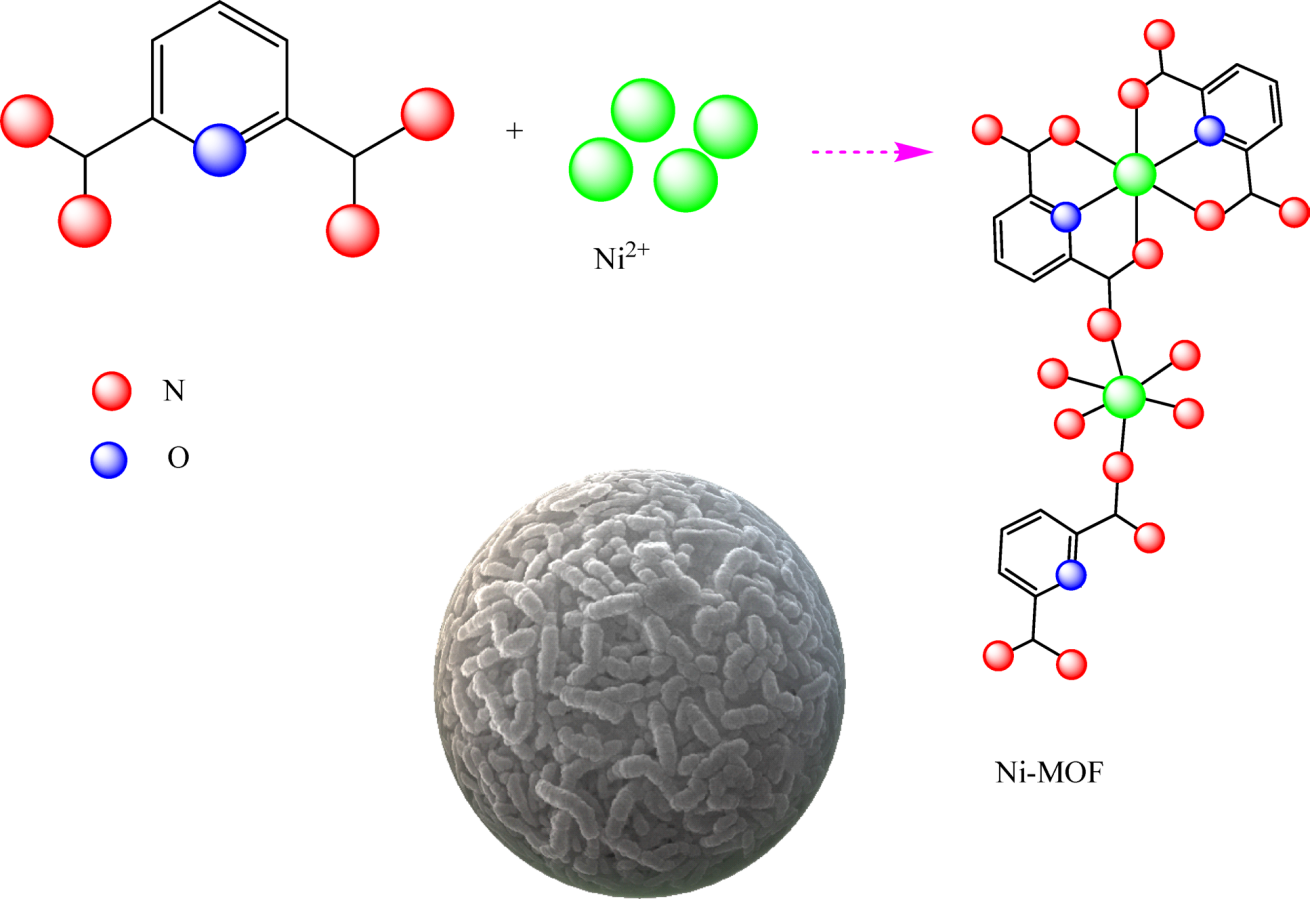
Diagrammatic representation of the synthesis of the Ni-MOF.

In a typical procedure, 2,6-Pyridinedicarboxylic acid (1 mmol) was dissolved in 5 mL of double-distilled water, followed by mixing 2 mmol of Ni (NO_3_)_2_.6H_2_O in dimethylformamide (20 mL). The mixture of metal and ligand was stirred for 10 min and then sonicated. Afterward, it was transferred into the autoclave at 160 °C for 15 h. The purple precipitate was formed. Then, the solid Ni-MOFs sample was filtered and washed with ethyl acetate. Finally, the precipitate was dried at 60 °C in a vacuum (Fig. 1).

### Synthesis of CoFe_2_O_4_/Ni-MOF

2,6-Pyridinedicarboxylic acid (1 mmol) was dissolved in 5 mL of double-distilled water, followed by mixing 2 mmol of Ni (NO_3_)_2_.6H_2_O in dimethylformamide (20 mL). Then, CoFe_2_O_4_ (1 g) was added. Afterward, the mixture was sonicated for 40 min and then placed into the autoclave at 160 °C for 15 h. Finally, the precipitate was dried at 60 °C in a vacuum.

### General process for the synthesis of 1*H*- tetrazoles

3 mL of H_2_O/EtOH was added to the mixture of nitrile (1 mmol), sodium azide (1.2 mmol), and catalyst (80 mg), and placed at 75 °C. After the end of the reaction, the catalyst was filtered, and the reaction mixture was treated with ethyl acetate and acidified with HCl (10 mL, 5 N). After that, 2–5 mL of cold water was added and washed several times. Finally, it was dried.

### Spectral data

5-(4-Nitrophenyl)−1*H*-tetrazole.

(Table 3, Entry 2)^1^:HNMR (400 MHz, DMSO, ppm): δ 8.50–8.53 (d, 2 H), 8.40–8.44 (d, 2 H).

**Table 1:** Catalytic performance of different catalysts for the synthesis of 5-substituted-1 H-tetrazole via a [3 + 2] cycloaddition reaction.

Entry	Catalyst	Catalyst loading (mg or mol%)	T (°C)	Solvent	Time (h)	Yield (%)	References
1	Boehmite@SiO_2_@Tris-Cu(I)	0.2 mol %	120	PEG (Poly (ethylene glycol)−400	120	89	^59,60^
2	BNPs@Cur-Ni	40 mg	120	PEG-400	75 min	97	^59,61^
3	Cu-Guanidine@BO-NPs	40 mg	120	PEG-400	70 min	95	^59,62^
4	Pd-SMTU @boehmite	20 mg	120	PEG-400	2.5	95	^59,63^
5	Pd-Arg@boehmite	25 mg	120	PEG-400	7	97	^59,64^
6	Pd-isatin@boehmite	35 mg	120	PEG-400	7	96	^65^
7	CoFe_2_O_4_@glycine-Yb	70 mg	120	PEG-400	2.33	93	^66^
8	Methionine@Fe_3_O_4_	50 mg	120	DMSO	0.166	100	^67^
9	Fe_3_O_4_@SiO_2_-APTES-TFA	100 mg	80	Ethanol	4	97	^68^
10	Cu-TBA@biochar	0.78 mol%	130	PEG	7	98	^69^
11	SBA-15@glycine-Cu	40 mg	100	PEG	110 min	98	^70^
12	Cu (II)-DCC-CMK-3	20 mg	120	PEG	90 min	97	^71^
13	Cu-MOFs-2 (Cu-TMU-17-NH_2_)	50 mg	110	PEG	5	95	^72^
14	SO_3_H@MCM-41	50 mg	80	DMF	1.6	90	^73^
15	Pd-SBT@MCM-41	35 mg	120	PEG-400	6.75	97	^74^
16	L-Cysteine@MCM-41	2.9 mol%	100	PEG-400	2.5	97	^75^
17	Ni-cytosine@MCM-41	0.1 mol%	120	PEG-400	1	92	^76^
18	Fe_3_O_4_@MCM-41-SB-Cu	30 mg	120	DMF	1	95	^77^
19	Cu-BTC frameworks	100 mg	130	PEG-600	120	93	^78^
20	Cu-MOFs-2 (Cu-TMU-17-NH_2_)	50 mg	110	PEG-400	5	95	^72^
21	Cu-MOF‐1	50 mg	110	PEG-400	5	94	^72^
22	Zn- MOF (Zn‐TMU‐17‐ NH_2_)	50 mg	110	PEG-400	60	55	^72^
23	Cu-MOF (Cu_2_(BDC)_2_(DABCO)	20 mg	120	DMF	60	93	^79^
24	Co-MOF (C0_2_(BDC)_2_(DABCO)	20 mg	120	DMF	120	79	^79^
25	Ni-MOF (Ni_2_(BDC)_2_(DABCO)	20 mg	120	DMF	120	51	^79^
26	Ni-MOF	80 mg	75	H_2_O/EtOH (1:1)	40	80	This Work
27	CoFe_2_O_4_/Ni-MOF	80 mg	75	H_2_O/EtOH (1:1)	20	80	This Work

## Results and discussion

### Catalyst characterization

#### N_2_ adsorption − desorption isotherms

**Fig. 2:**
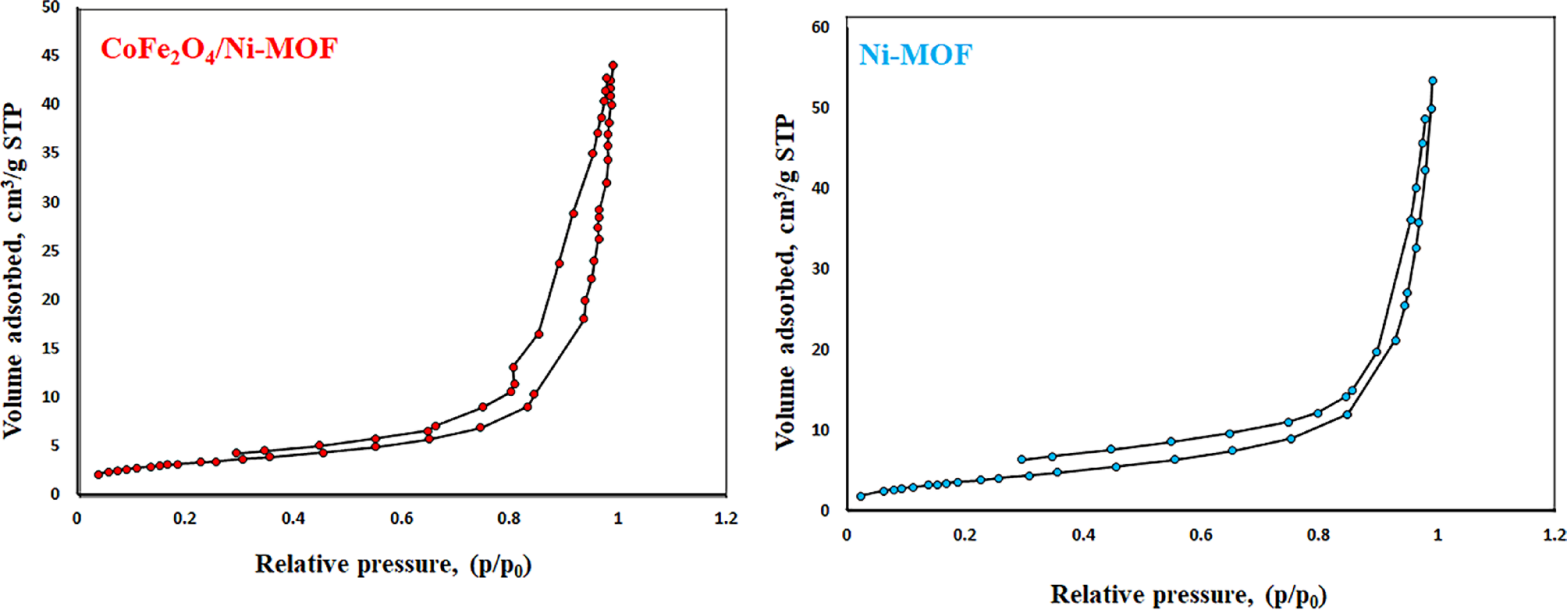
N_2_ adsorption − desorption isotherms of the CoFe_2_O_4_/Ni-MOF, and the Ni-MOF.

**Fig. 3:**
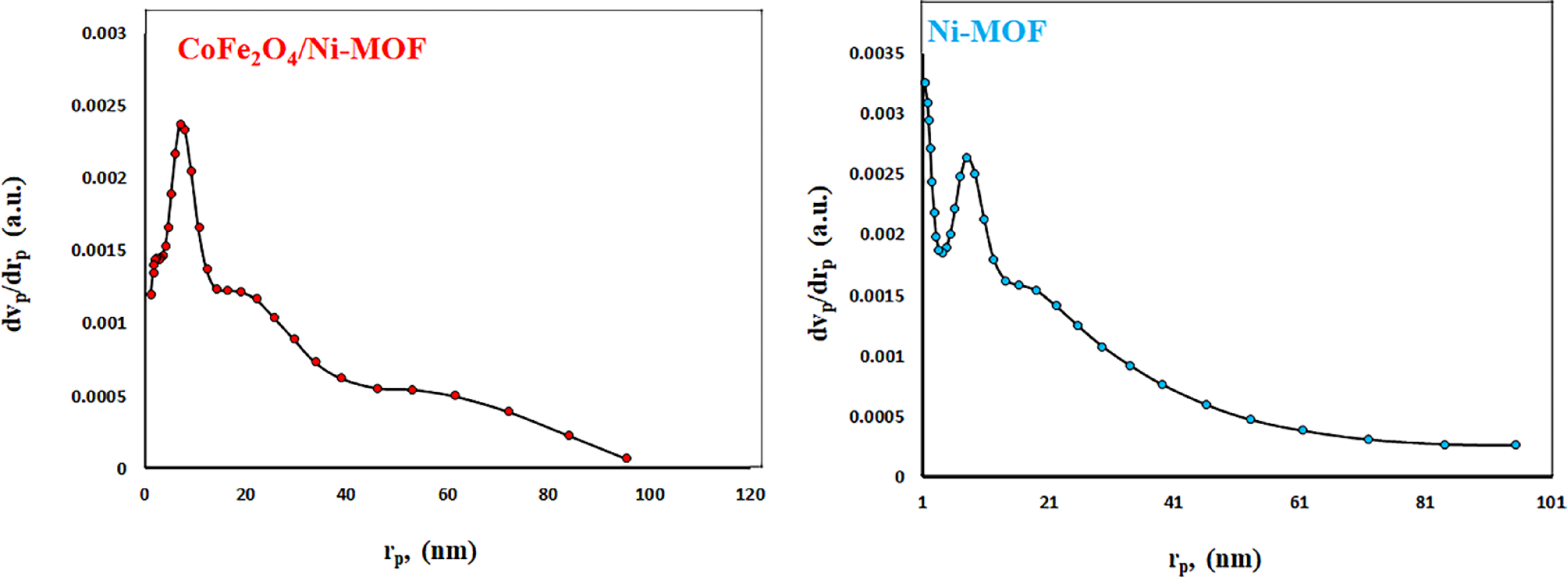
BJH pore width distribution of the CoFe_2_O_4_/Ni-MOF, and the Ni-MOF.

The BET and t-plot were used to measure the surface area and the BJH method of the CoFe_2_O_4_/Ni-MOF and Ni-MOF (Figs. 2 and 3). The surface area by the Brunauer-Emmett-Teller (S_BET_) method, average pore sizes (d_avg_), and total pore volumes (V_total_) of the CoFe_2_O_4_/Ni-MOF and Ni-MOF are shown in Table 2. The H3 hysteresis loop for a type IV adsorption-desorption isotherm indicates the synthesized composite has a mesoporous structure (Fig. 1)^80^. Based on the given information, it can be observed that the surface area (S_BET_) can be calculated as 11.39 m^2^/g (CoFe_2_O_4_/Ni-MOF) and 14.23 m^2^/g (Ni-MOF). Also mean pore size is 23.59 nm (CoFe_2_O_4_/Ni-MOF), and 22.28 nm (Ni-MOF). Pore volume is 0.067 cm^3^/g (CoFe_2_O_4_/Ni-MOF) and 0.079 cm^3^/g (Ni-MOF).

**Table 2:** The textural characteristics of synthesized samples.

Entry	Catalyst ^b^	S_BET_ (m^2^/g)	d_avg_ (nm)	V _total_ (cm^3^/g)
1	CoFe_2_O_4_/Ni-MOF	11.39	23.59	0.067
2	Ni-MOF	14.23	22.28	0.079

#### FE-SEM and EDX analysis

**Fig. 4:**
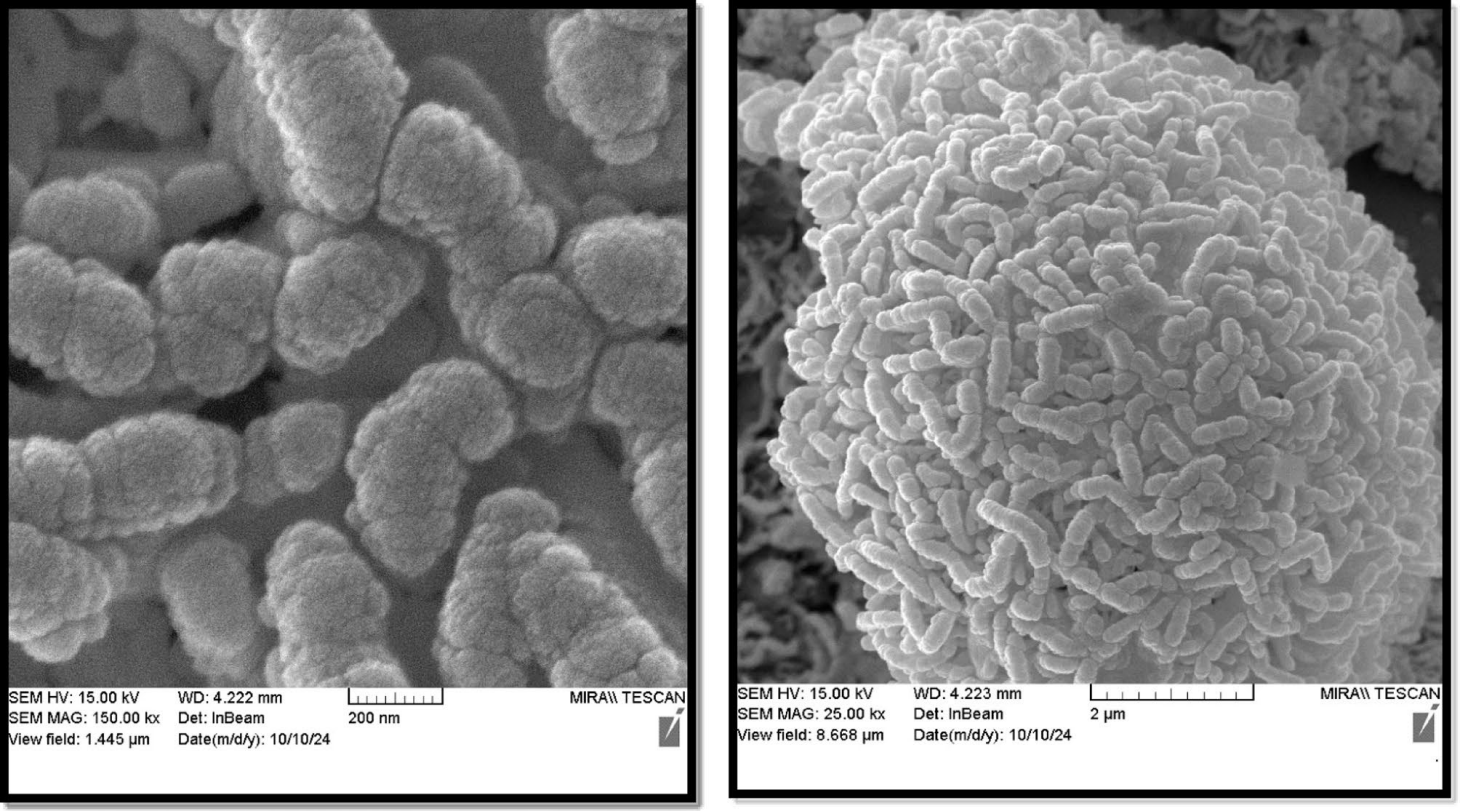
SEM images of the Ni-MOF.

**Fig. 5:**
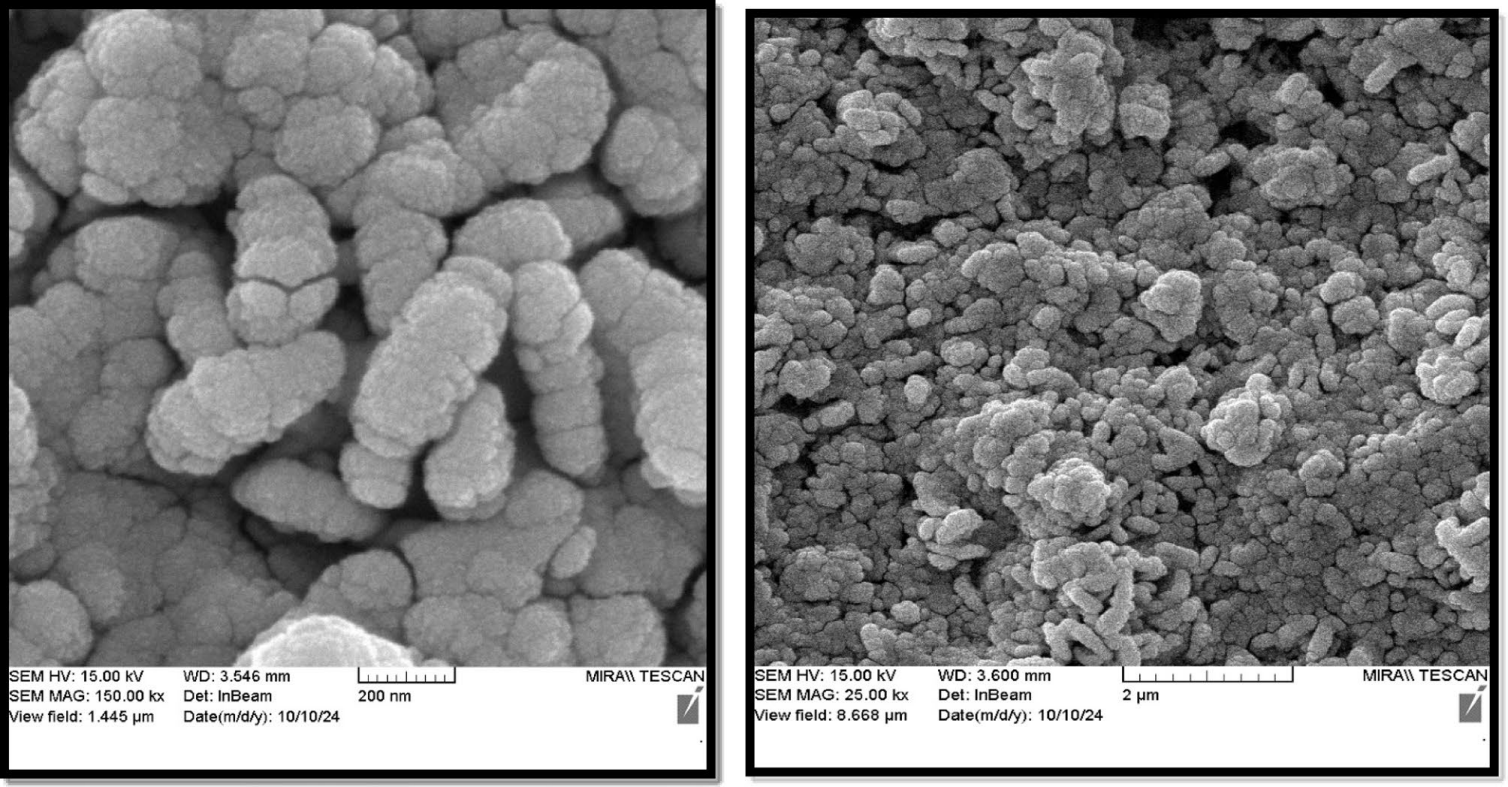
SEM images of the CoFe_2_O_4_/Ni-MOF.

FE-SEM was used to examine the dimensions, size, shape, and textural morphology of the CoFe_2_O_4_/Ni-MOF and Ni-MOF. The images at various magnifications are shown in Figs. 4 and 5. The particles are homomorphic and worm-shaped. The apparent morphology is not significantly altered by the CoFe_2_O_4_ association. The CoFe_2_O_4_ association has caused reduced agglomeration of the Ni-MOF particles.

**Fig. 6:**
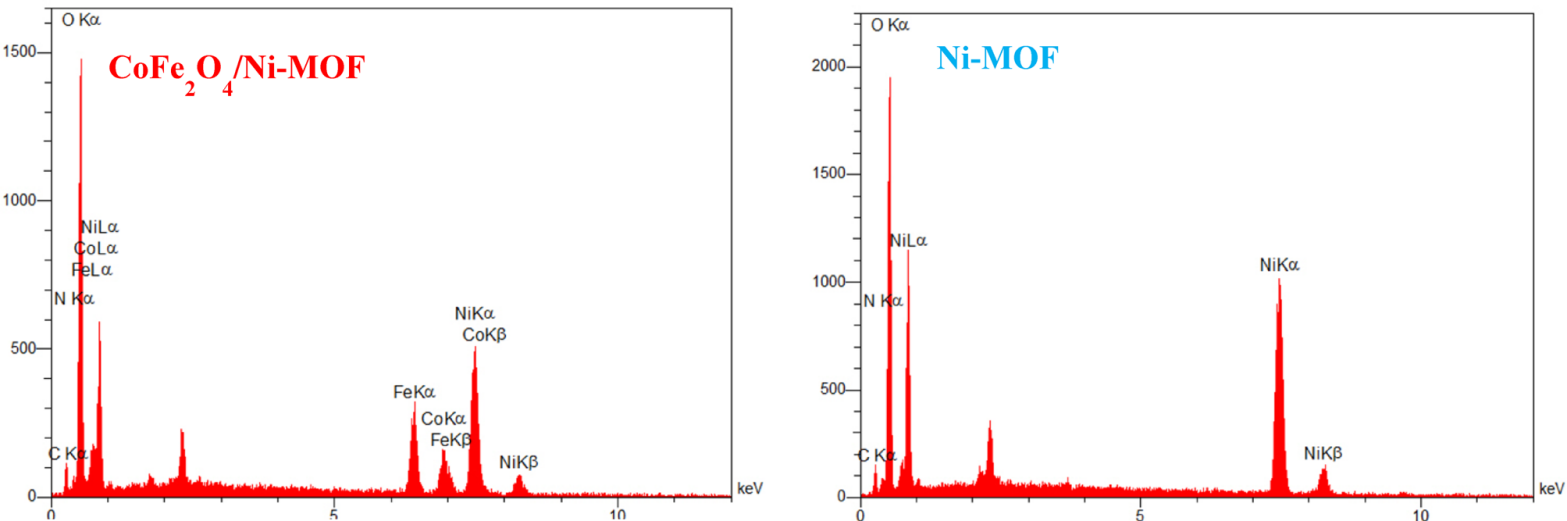
EDX of the CoFe_2_O_4_/Ni-MOF, and Ni-MOF.

The EDX spectrum of CoFe_2_O_4_/Ni-MOF and Ni-MOF (Fig. 6) shows iron (Fe), cobalt (Co), nickel (Ni), carbon (C), nitrogen (N), and oxygen (O), indicating successful synthesis of the hybrid composite.

#### Magnetic properties

**Fig. 7:**
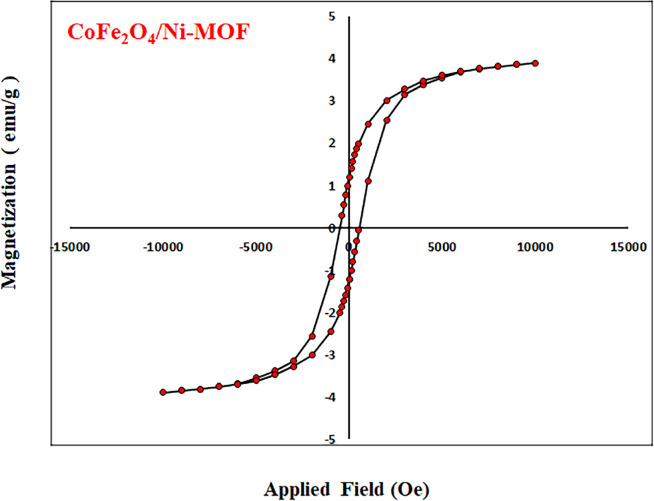
Magnetization curves of the CoFe_2_O_4_/Ni-MOF.

The magnetic properties of the CoFe_2_O_4_/Ni-MOF composite are critical for effectively recovering magnetic from solution-based processes. The magnetic properties of the CoFe_2_O_4_/Ni-MOF composite were measured using a vibrating sample magnetometer, as shown in Fig. 7. Spinel ferrites have the general formula [B^3+^] O^2−^} (A^2+^)} where A^2+^ and B^3+^ are divalent and trivalent cations, respectively. A ferrimagnetic material is defined as one in which the magnetic dipole of the atoms on different sublattices are opposed as in antiferromagnetism, but in ferrimagnetic materials, opposing moments are unequal, and a spontaneous net magnetization remains. The magnetic dipole moments in a ferrimagnetic material are divided into sublattices. Each sublattice can be treated as a ferromagnetic material, and the difference between the magnetic dipole moments for the sublattices results in the net magnetization for the ferrimagnetic materials. This happens when the sublattices consist of different materials or ions, such as M^2+^ and M^3+^ in ferrites^81^. The saturation magnetization of CoFe_2_O_4_/Ni-MOF has a saturation magnetization of 3.9 emu g^−1^ and remnant magnetization of 1.21 emu/g, but Ni-MOF has no magnetic characteristics.

#### XRD analysis

**Fig. 8:**
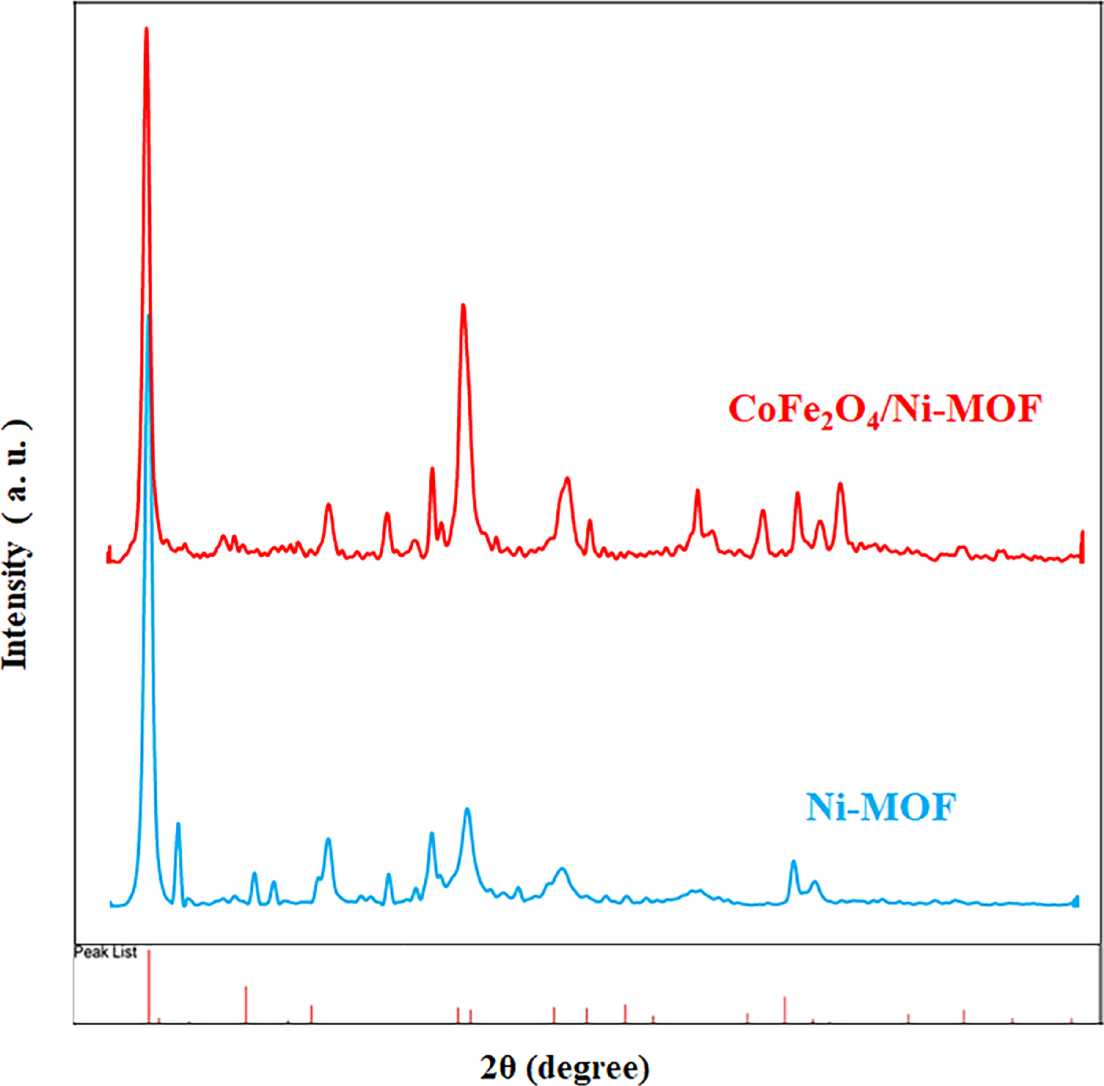
XRD patterns of the CoFe_2_O_4_/Ni-MOF and the Ni-MOF.

XRD analysis was conducted to assess the structural integrity and crystallinity of the produced materials (Fig. 8). The XRD pattern of CoFe_2_O_4_ displays discrete diffraction peaks at rounds 18.3, 30.2, 35.6, 43.0, 53.5, 57.1, and 62.7°, which are attributed to the spinel ferrite type CoFe_2_O_4_ (JCPDS No. 22–1086) (111), (220), (311), (400), (422), (511), and (440) planes^82^. The XRD pattern of CoFe_2_O_4_/Ni-MOF and Ni-MOF showed diffraction peaks at rounds 12.0, 15.0, 20.0, 22.0, 25.0, 33.0, and 37.0°^42^.

#### FT-IR analysis

**Fig. 9:**
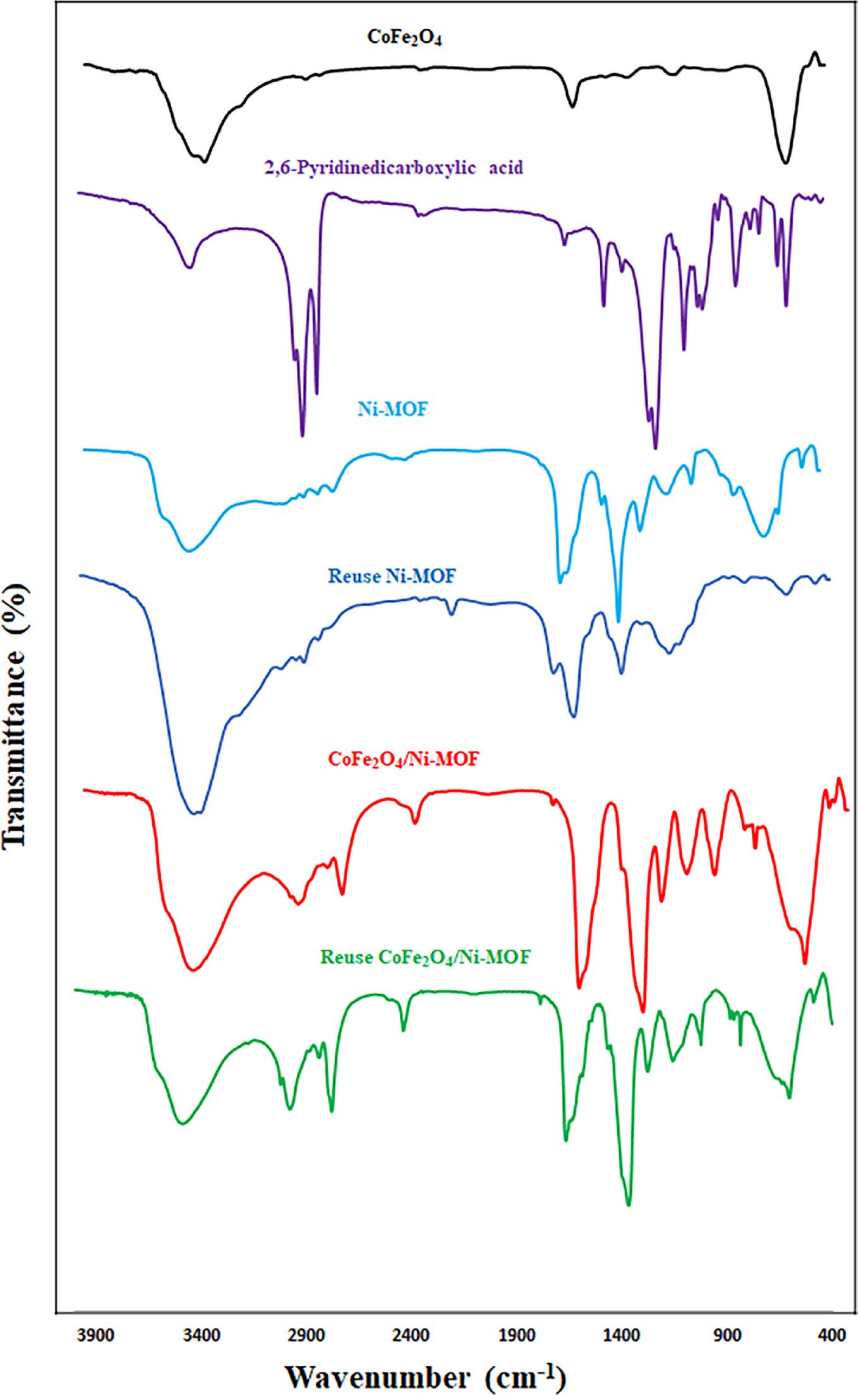
FT-IR spectra of CoFe_2_O_4_, 2,6-Pyridinedicarboxylic acid, Ni-MOF, reuse Ni-MOF, CoFe_2_O_4_/Ni-MOF and reuse CoFe_2_O_4_/Ni-MOF.

The infrared spectra of the CoFe_2_O_4_, 2,6-Pyridinedicarboxylic acid, Ni-MOF, reused Ni-MOF, CoFe_2_O_4_/Ni-MOF, and reused CoFe_2_O_4_/Ni-MOF samples were investigated, and they can be seen in Fig. 8. The tetrahedrally complexed trivalent metal-oxygen (Fe-O) and octahedrally complexed bivalent metal-oxygen (Co-O) are represented by the bands that appeared at 587 cm^−1^ and 492 cm^−1^, respectively^83^. The band at around 3200–3700 cm^−1^ is related to the OH groups of H-O-H or surface hydroxyl group stretching vibrations of the CoFe_2_O_4_. The band in the region of 1623–1650 cm^−1^ is due to the H-O-H bending vibration^84^. The two bands at 2922 and 2850 cm^−1^ are related to the C–H stretch of 2,6-Pyridinedicarboxylic acid^85^. The FTIR spectra of compounds 2,6-Pyridinedicarboxylic acid, Ni-MOF, and CoFe_2_O_4_/Ni-MOF show symmetric stretching vibrations of the carboxylate groups of 2,6-pyridine dicarboxylate moieties at around 1382 cm^−1^^86^. A band at 1668 cm^−1^ may be due to the amide carbonyl group of DMF. The absorption peaks at 1593, 1083, and 765 cm^−1^ in the FTIR spectrum are caused by -NH stretching, C-N stretching, and -CH_2_ rocking vibrations of coordinated moieties, respectively. The nature of the recovered catalyst was investigated by FT-IR (Fig. 9). FT-IR spectra of the recovered catalyst indicated that the catalyst can be recycled four times without a significant change in the structure of the Ni-MOF and CoFe_2_O_4_/Ni-MOF.

### Catalyst evaluation

The potential of Ni-MOF and CoFe_2_O_4_/Ni-MOF as catalysts was investigated for the one-pot conversion of nitrile to tetrazole using benzonitrile (1 mmol), catalyst, and NaN_3_ (1.2 mmol) at different temperatures in different solvents. To optimize the reaction conditions, the efficiency of catalyst amounts, various solvents, and different temperatures has been studied.

Different solvents such as EtOH, EtOH/water, and water were tested. In EtOH solvent, the reaction yield was 30% in 40 min (Table 3, entry 1). The reaction yield in EtOH/water (1:1) solvent is 80% in 40 min (Table 3, entry 2). The reaction yield in water solvent is 40% in 40 min (Table 3, entry 3). Accordingly, EtOH/water (1:1) solvent was selected as the intended solvent for this method. After choosing the solvent, different temperatures of 75, 60, and 40 ℃ were tested, based on Table 3 in EtOH/water (1:1) solvent at 75 ℃, for 40 min, the reaction yielded 80%. (Table 3, entry 2), and at a temperature of 60 °C in 55 min, the reaction yielded 55% (Table 3, entry 5), and at 40 °C in 95 min, the reaction yielded 43% (Table 3, entry 6). So, a temperature of 75 ℃ was chosen to continue the reaction process. After choosing the best solvent, the amount of catalyst was changed according to Table 3, and the value of 80 mg of catalyst was chosen. From Table 3, entry 10, the CoFe_2_O_4_/Ni-MOF shows better activity in the synthesis of 5-substituted 1*H*-tetrazoles (the reaction yield in H_2_O/EtOH (1:1) solvent with 80 mg of catalyst is 80% in 20 min) in comparison with the Ni-MOF sample (the reaction yield in H_2_O/EtOH (1:1) solvent with 80 mg of catalyst is 80% in 40 min).

Using the optimized conditions, several tetrazole derivatives have been prepared with Ni-MOF, which can be seen in Table 4. In this research, different combinations of aromatic nitriles with electron-withdrawing and electron-donating groups have been used. The ability to achieve successful conversion in a short time is a significant advantage of this method.

**Fig. 10:**
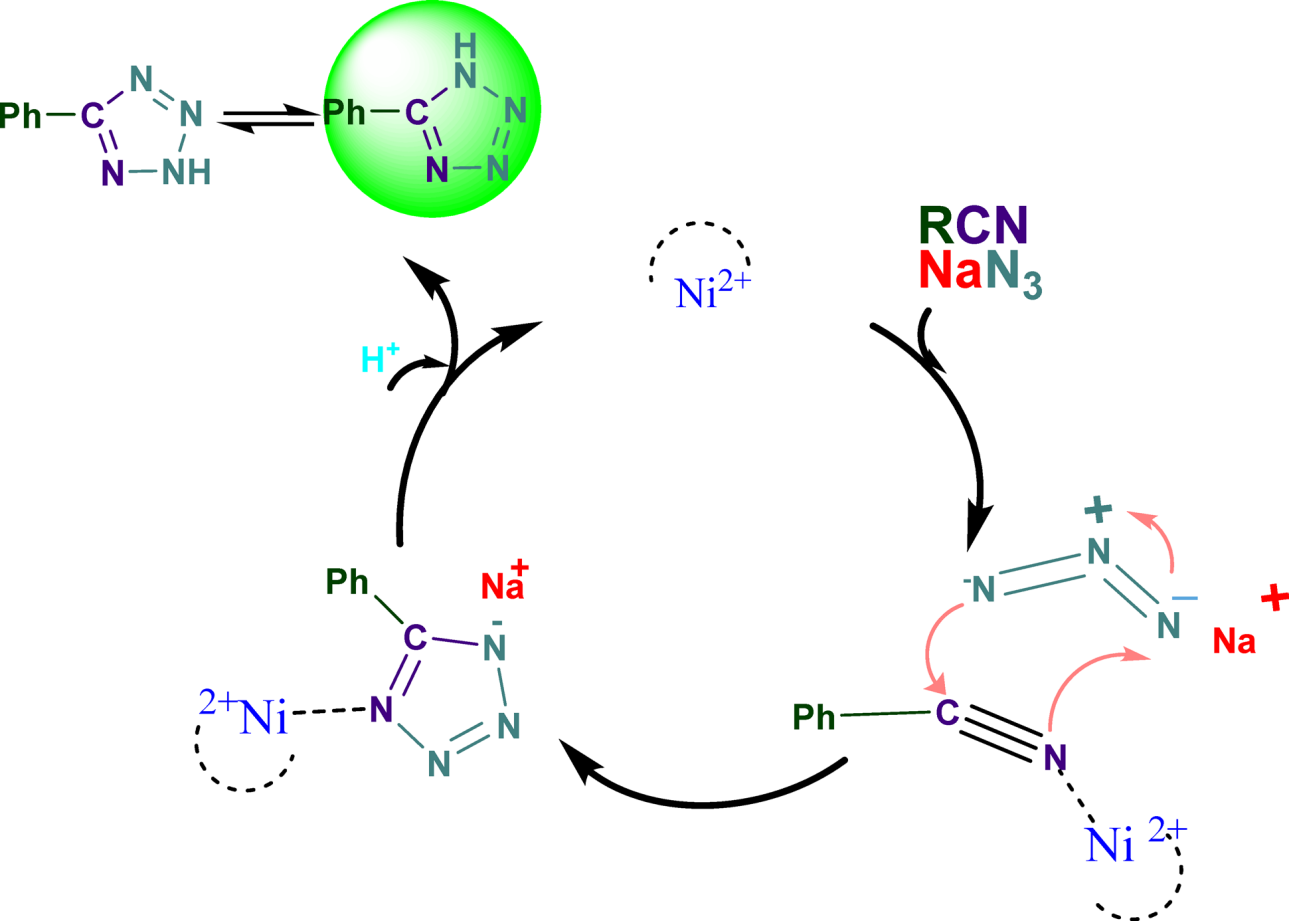
Plausible reaction pathway for the direct conversion over the Ni-MOF Catalyst.

The formation of 1*H*-tetrazoles depends critically on the nitrile coordination of the substrate with the Lewis acidic Ni (II). The Lewis acidic Ni (II) acts as an active site for the coordination of the nitrile molecule. The primary element affecting 1,3-dipolar cycloadditions is the coordination of Ni (II) to the nitrile. Tetrazole is the final result of further hydrolysis and azide nucleophilic assault. When the nitrile substrate coordinates with Ni^2+^, it forms a stable complex that activates the nitrile toward nucleophilic attack. This coordination increases the electrophilicity of the carbon atom in the nitrile, making it more susceptible to nucleophilic attack by azide ions^87^ (Fig. 10).

**Table 3:** 1*H*-tetrazole yields at different solvents, catalyst amounts, and temperatures in the presence of a catalyst.^a^.

Entry	Catalyst	Solvent	Catalyst (mg)	Temperature (^°^C)	Time (min)	Yield (%)^b^
1	Ni-MOF	EtOH	80	75	40	30
2	Ni-MOF	H_2_O/EtOH (1:1)	80	75	40	80
3	Ni-MOF	H_2_O	80	Boiling point	40	40
4	Ni-MOF	H_2_O/EtOH (1:1)	80	60	55	55
5	Ni-MOF	H_2_O/EtOH (1:1)	80	40	95	43
6	Ni-MOF	H_2_O/EtOH (1:1)	100	75	40	82
7	Ni-MOF	H_2_O/EtOH (1:1)	60	75	40	45
8	Ni-MOF	H_2_O/EtOH (1:1)	40	75	40	28
9	-	H_2_O/EtOH (1:1)	-	75	24 h	nil
10	CoFe_2_O_4_/Ni-MOF	H_2_O/EtOH (1:1)	80	75	20	80

^a^Reactions conditions: Nitrile (1 mmol), NaN_3_ (1.2 mmol, 0.078 g), catalyst, and solvent (3 mL) at a temperature.

^b^Isolated yield.

**Table 4:** Synthesis of 5-substituted 1*H*-tetrazole derivatives in the presence of Ni-MOF.^a^.

Entry	Substrate	Product	Time (min)	Yield (%)	M. p (^°^C)
1			40	45	150–151 ^61^
2			40	30	217–219 ^61^
3			60	65	182–184
4			60	75	262–265 ^47^
5			40	80	213–215 ^61^
6			70	50	121–122 ^47^
7			100	50	233–235 ^88^
8			80	30	180–182 ^61^
9			80	60	262–264 ^61^
10			80	50	130–133 ^47^

^a^Reactions conditions: Nitrile (1 mmol), NaN_3_ (1.2 mmol), H_2_O/EtOH (1:1), (3 mL), and catalyst (80 mg) at 75 ℃.

### Catalyst reusability

**Fig. 11:**
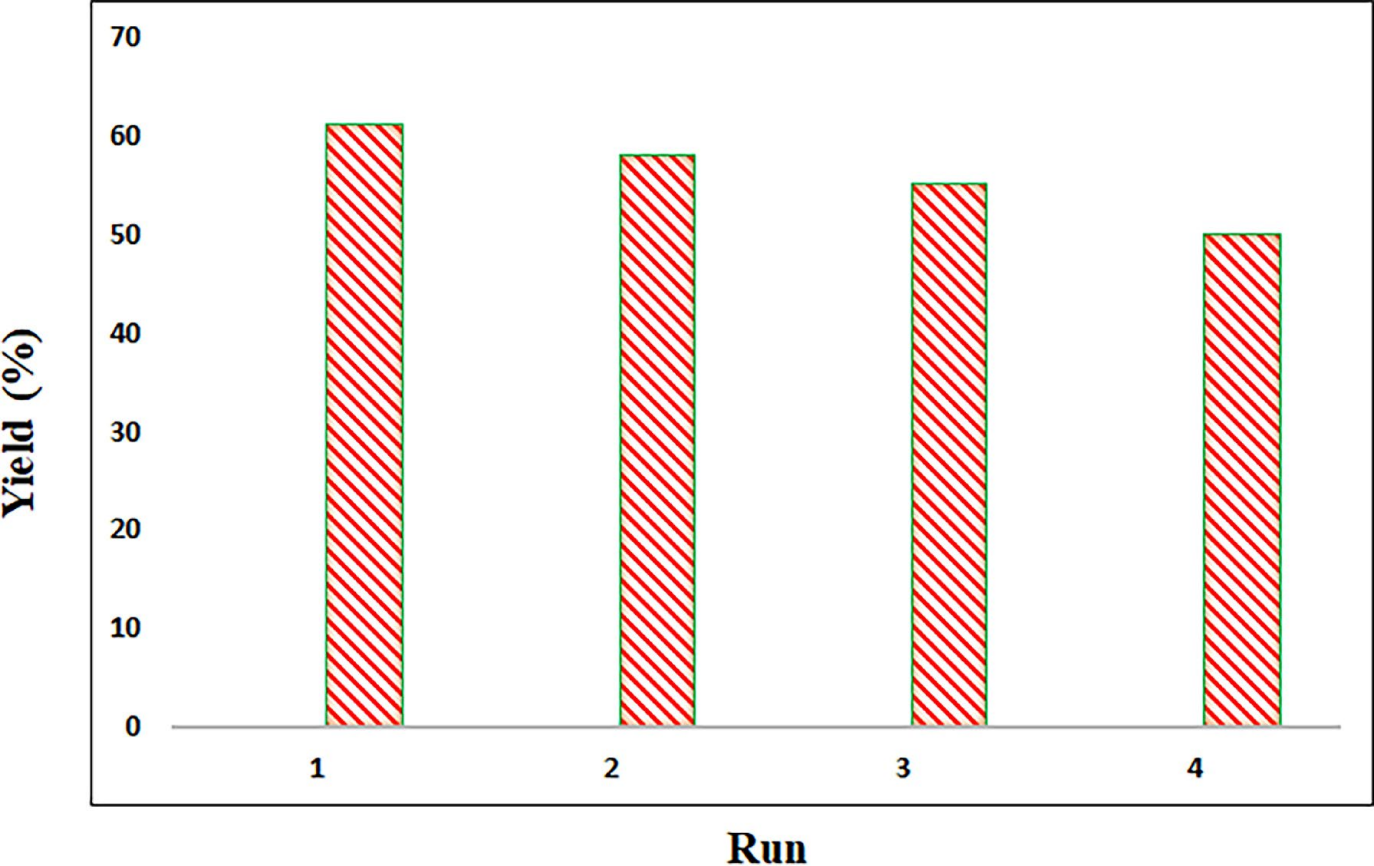
Reusability of the Ni-MOF Catalyst.

To check the possibility of recycling the Ni-MOF catalyst for the one-pot conversion of nitrile to tetrazole with malononitrile (1 mmol), catalyst (80 mg), and NaN_3_ (1.2 mmol), in EtOH/water (1:1). The catalyst was separated and washed with ethyl acetate, and this recycled catalyst was used in the new reaction. The results for the heterogeneous catalyst for the synthesis of tetrazole are shown in Fig. 11. As can be seen, there is no significant reduction in the conversion of nitrile to tetrazole after the 4 cycles for Ni-MOF.

## Leaching study

The leaching of the catalyst was investigated with ICP-AES (Inductively coupled plasma atomic emission spectroscopy). ICP-AES analysis of the fresh and recovered catalyst was investigated. Using the ICP-AES technique, the Ni quantity of the catalyst before and after the reaction for the preparation of 5-(4-chlorophenyl)−1*H*-tetrazole was determined. It was obtained that the quantity of Ni in the fresh Ni-MOF catalyst is 5.31 mmol g^−1^. Ni content has slightly decreased to 5.0 mmol g^−1^ after the 4th catalytic reaction cycle.

## Conclusion

The Ni-MOF and CoFe_2_O_4_/Ni-MOF as two novel heterogeneous nanocomposite catalysts were synthesized using 2,6-Pyridinedicarboxylic acid and nickel nitrate. The catalysts were confirmed by different characterization techniques such as BET, XRD, SEM, EDX, VSM, and FT-IR. FE-SEM images show that the particles are homomorphic and worm-shaped. Crucially, the association with CoFe₂O₄ reduced agglomeration of Ni-MOF particles without significantly altering the overall worm-like morphology. This is a key improvement as less agglomeration often leads to more accessible active sites. The Type IV isotherm with an H3 hysteresis loop confirms a mesoporous structure for both materials, which is beneficial for mass transfer in catalytic reactions. The BET data show that adding CoFe₂O₄ slightly decreases the surface area (14.23 → 11.39 m²/g) and pore volume (0.079 → 0.067 cm³/g) while slightly increasing the pore size (22.28 → 23.59 nm). This is consistent with the incorporation of magnetic nanoparticles within/on the MOF pores. The VSM data show that Ni-MOF shows no magnetic character, while CoFe₂O₄/Ni-MOF has a saturation magnetization (Ms) of 3.9 emu/g. Although this is a relatively low value, it is likely sufficient to allow for convenient separation of the catalyst from the reaction mixture using an external magnet, which is a major advantage for a heterogeneous catalyst. Both catalysts are effective, but the magnetic composite (CoFe₂O₄/Ni-MOF) shows superior catalytic performance for the synthesis of 5-substituted 1 H-tetrazoles under mild, green conditions (75 °C, H_2_O/EtOH (1:1) solvent), achieving an 80% yield in 20 min versus 40 min for the Ni-MOF, using the same catalyst mass (80 mg). There is no significant reduction in the conversion of nitrile to tetrazole after the 4 cycles for the Ni-MOF catalyst. This indicates the structure of catalyst remains intact and active sites are preserved throughout the reaction and work-up process, a vital feature for practical applications.

## Data Availability

The datasets used and analyzed during the current study are available from the corresponding author upon reasonable request.
